# Impact of wave whitecapping on land falling tropical cyclones

**DOI:** 10.1038/s41598-017-19012-3

**Published:** 2018-01-12

**Authors:** Nicolas Bruneau, Ralf Toumi, Shuai Wang

**Affiliations:** 1 Blackett Laboratory, Department of Physics, Imperial College, Prince Consort Road, London, SW7 2AZ UK; 2 Present Address: National Oceanography Centre, Joseph Proudman Building, Liverpool, L3 5DA UK

## Abstract

Predicting tropical cyclone structure and evolution remains challenging. Particularly, the surface wave interactions with the continental shelf and their impact on tropical cyclones have received very little attention. Through a series of state-of-the-art high-resolution, fully-coupled ocean-wave and atmosphere-ocean-wave experiments, we show here, for the first time, that in presence of continental shelf waves can cause substantial cooling of the sea surface. Through whitecapping there is a transfer of momentum from the surface which drives deeper vertical mixing. It is the waves and not just the wind which become the major driver of stratified coastal ocean ahead-of-cyclone cooling. In the fully-coupled atmosphere-ocean-wave model a negative feedback is found. The maximum wind speed is weaker and the damaging footprint area of hurricane-force winds is reduced by up to 50% due to the strong wave induced ocean cooling ahead. Including wave-ocean coupling is important to improve land falling tropical cyclone intensity predictions for the highly populated and vulnerable coasts.

## Introduction

Tropical cyclones (TCs) are one of the most damaging natural catastrophes. Due to intense destructive winds and heavy rainfall associated with storm surges, large waves and flooding, TCs are a major threat to human lives and properties. Therefore, accurately forecasting their structure and track is crucial to be able to provide useful warnings, to improve global resilience and to preserve public confidence in forecasts. Although significant progress has been achieved over the past four decades^1^ (http://www.nhc.noaa.gov/verification/verify5.shtml), particularly reducing track errors, cyclone intensity forecasts have seen no large improvements. These limitations have been attributed to atmospheric model deficiencies (boundary layer dynamics, grid resolution, for example) as well as the misrepresentation of the ocean dynamics and state^2–4^.

Air-sea heat and moisture exchanges are two key processes in TC intensification^2,5^ and are directly linked to ocean surface cooling/warming due to changes of mixed layer depth (MLD) and upwelling/downwelling^6^. Therefore, ocean models need to accurately predict the rate and pattern of sea surface temperature (SST) cooling^7^ and the upper-ocean characteristics more generally. Over the past decade the ocean-atmosphere interface has received much attention^8–13^ due to its major role in TC dynamics. Particularly, modulation of TC intensity can be significantly driven by the vertical structure of the ocean^9^ and SST negative feedbacks can substantially diminish the TC intensity^8^. More recently and within the context of TC intensification, TC and mesoscale oceanic features (eddies) interactions^14,15^ as well as Langmuir-induced turbulence^6^ have been investigated. Large ahead-of-cyclone-eye cooling over a strongly-stratified continental shelf was reported during Hurricane Irene (2011) and such cooling was present for all cyclones reaching the Mid-Atlantic Bight continental shelf as well as in other regions with TC activity across the world^16,17^.

A growing interest in wave interactions with ocean and atmosphere has recently emerged^18–23^. Wave-induced dynamics are now considered a key process driving the complex climate system by exchanges at the ocean-atmosphere interface^24–27^, modifying both atmospheric boundary layer the and upper-ocean dynamics. “[…] Such models, in spite of the apparent progress in their development, seem to have been reaching saturation in their performance and still unable to reproduce observed air-sea interaction phenomena such as the ENSO cycle and tropical cyclone intensity, among others. There is an apparent need for additional physics for such models, and coupling with the waves does offer such physics”^25^. Due to their wide extension and intense winds, TCs can be a considerable source of large waves^28–30^. Recent studies^31–33^ emphasize that realistic SST and MLD patterns are necessary to accurately model TC characteristics both over deep oceans and in coastal regions. While the impact of enhanced mixing due to waves has been investigated in deep water^23,30^, the impacts of waves in coastal regions, particularly the potential impact of wave-current interactions, before and during TC landfall have been mainly unexplored to date.

The present study focuses on the effects of wave-induced processes (both currents and mixing) on the stratified coastal ocean, in the presence of a continental shelf before a cyclone makes landfall. Particularly, the transfer of energy due to strong dissipation by whitecapping induced by the continental shelf is investigated. Through a set of high-resolution idealized ocean-only, ocean-wave and atmosphere-ocean-wave coupled simulations, we show for the first time the potential substantial surface cooling by wave-current interactions generated by a TC and a negative feedback on the TC.

## Results

### No wave simulations

The three-dimensional ocean response to TC is simulated using the Regional Ocean Modeling System^34^ (ROMS) for either an idealised deep ocean (noted *DEEP*) or a deep ocean combined with a continental shelf (*CSHF* - Supplementary Fig. S1). Spatially constant stratified ocean with a shallow MLD is used as the initial state. The ocean is forced with a moving parametric TC wind model^35^.

**Figure 1:**
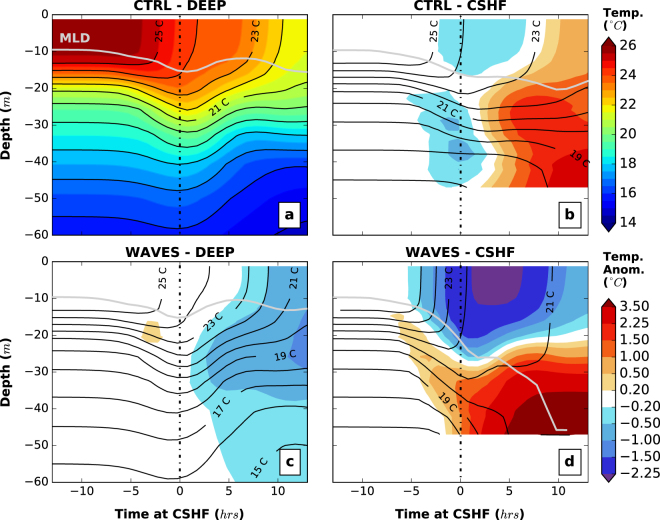
Ahead-of-the-cyclone-eye ocean cooling and anomaly time series. (**a**) For the deep water experiment with no wave-induced physics (*CTRL-DEEP*), (**b**) with the presence of a continental shelf (*CTRL-CSHF*), (**c**) and (**d**) same as (**a**) and (**b**) but accounting for wave-induced dynamics, *WAVES-DEEP* and *WAVES-CSHF*, respectively. The profile is averaged in an about 100−*km* square box (see Fig. 2b). (**b**,**c**,**d**) show the temperature profile anomalies to the reference case (**a**). The gray line represents the mixed layer depth (MLD) evolution and the temperature contours are provided every 1 *°C* in plain black lines. The 0 on the x-axis represents the time the cyclone eye reaches the continental shelf (i.e. negative values means ahead-of-the-cyclone-eye). The four simulations were carried out for an idealized analytical temporally-constant wind field characterized by a maximum wind speed *V*_*max*_ = 35 *m*/*s*, a radius of maximum wind *R*_*max*_ = 100 *km*, a transitional speed *U*_*bck*_ = 5 *m*/*s* and an ocean state with a *MLD* = 10 *m*, a continental shelf of 50 *m*– depth and about 200 *km*– wide with the *Weak* temperature profile (see *Methods* section for more details).

**Figure 2:**
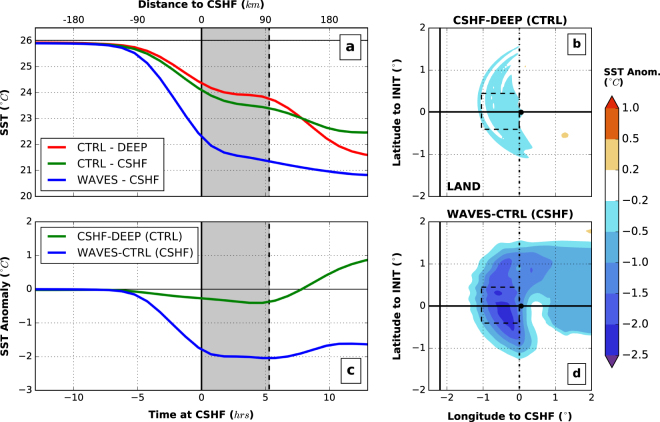
Impact of wave-induced dynamics on SST. (**a**) Spatially-averaged SST time series for the *CTRL-DEEP* (red), the *CTRL-CSHF* (green) and *WAVES-CSHF* (blue) experiments. (**b**) Averaged SST anomaly time series comparing the impact of the continental shelf without waves (*CSHF-DEEP (CTRL)* - green) and the impact of waves in the presence of a continental shelf (*WAVES-CTRL (CSHF)* - blue). The x-axis 0 represents the time the cyclone eye reaches the continental shelf. The gray bands illustrate when the cyclone is crossing the area where averages are computed (dashed box in **c** and **d**). The SST anomaly maps just before the cyclone reaches the continental shelf (black circles representing the eye) are presented in (**c**) and (**d**) for *CSHF-DEEP (CTRL)* and *WAVES-CTRL (CSHF)*, respectively. The cyclone characteristics and initial ocean state are the same than in Fig. 1.

The spatially-averaged vertical temperature profile evolution shows a cooling of about 1.6 *°C* of the ocean surface layer ahead-of-the-cyclone-eye as well as a deepening of the MLD due to the strong winds blowing ahead of the cyclone (Fig. 1a - in deep water without waves - *CTRL-DEEP* experiment). The cooling intensifies after the cyclone passage due to the presence of the cold wake behind of up to 4 *°C*. The described two modes clearly appear in the SST temporal evolution (Fig. 2a) with an initial cooling occurring ahead-of-the-cyclone-eye, a plateau when the cyclone eye is above the region of interest (gray area in Fig. 2a corresponding to the cyclone eye being over the continental shelf in Fig. 2b) and a second cooling induced by the wind behind the cyclone.

In the presence of a continental shelf, the cooling ahead is slightly stronger than in deep water with anomalies to the control simulation of only around 0.3 *°C* (15–20% - Fig. 1b - *CTRL-CSHF*) while the post-cyclone state is actually cooler in *CTRL-DEEP*. The MLD deepens to reach up to 20 *m*. The SST before the cyclone enters the region of interest shows a relatively homogeneous and weak enhancement of the cooling ahead of the cyclone induced by the continental shelf (Fig. 2b,c) which reduces onshore surface currents and enhances offshore bottom currents.

### With wave simulations

To take into account of the wave-induced processes, ROMS (Regional Ocean Modeling System) is coupled^36,37^ to the third-generation spectral wave model SWAN^38^ (Simulating WAves Nearshore). In deep water, the waves have a negligible impact on the ahead-of-the-cyclone cooling (Fig. 1c - *WAVES-DEEP*) even with modelled significant wave height (*H*_*s*_) reaching 13 *m*. The cold wake is intensified around 6–10 hours after the cyclone crosses the region (anomalies to control simulation presented in Fig. 1c). The model does not directly account for an enhanced shear production or eddy viscosity/diffusivity due to waves^23,39^. This explains the weak impacts of the wave-induced processes on TC evolution for the deep ocean due to ocean-wave coupling as wave-current interactions remain weak and the only injection of turbulence at the surface due to waves is not enough to break the stratification. However, on the continental shelf, the wave impact increases dramatically. The ahead-of-the-cyclone-eye cooling becomes significantly enhanced (Fig. 1d - *WAVES-DEEP*). The cooling is an order of magnitude stronger than the *CTRL-CSHF* experiment (Table 1). The bottom layer of the ocean also warms in response to the surface mixing within a few hours delay. Ahead-of-the-cyclone-eye the MLD reveals a strong deepening (reaching 23 *m*) and this deepening is amplified later with the winds behind the cyclone; after about 10 hours, the entire water column over the continental shelf is almost mixed. Over 75% of the SST cooling occurs ahead-of-the-cyclone-eye (Fig. 2a–c) and while a small asymmetry towards the left of the track is present in the SST anomalies compared to *CTRL-CSHF* (Fig. 2d), the global cooling pattern ahead of the cyclone remains homogeneous and much larger than in the wind only case.

**Table 1:** Sensitivity analysis: rounded averaged cooling ahead-of-the-cyclone-eye (and % ratio to total cooling).

Experiment N°	1	2	3	4	5	6	7	8	9	10	11
*V*_*max*_ (*m*/*s*)	**25**	**45**	35	35	35	35	**35**	35	35	35	35
*R*_*max*_ (*km*)	100	100	**50**	100	100	100	**100**	100	100	100	100
*U*_*bck*_ (*m*/*s*)	−5	−5	−5	−**3**	−**7**	−5	−**5**	−5	−5	−5	−5
Direction (°)	0	0	0	0	0	**45**	**0**	0	0	0	0
CSHF (*m*)	50	50	50	50	50	50	**50**	**80**	50	50	50
MLD (*m*)	10	10	10	10	10	10	**10**	10	**25**	10	10
*T* Profile	Weak	Weak	Weak	Weak	Weak	Weak	**Weak**	Weak	Weak	**Steep**	Weak
*f*_*coriolis*_ at	35 °*N*	35 °*N*	35 °*N*	35 °*N*	35 °*N*	35 °*N*	**35** °*N*	35 °*N*	35 °*N*	35 °*N*	**20** °*N*
DEEP (CTRL)	0.3 °*C*	4.1 °*C*	0.5 °*C*	2.2 °*C*	1.3 °*C*	1.2 °*C*	**1.6** °*C*	1.6 °*C*	0.2 °*C*	1.9 °*C*	1.9 °*C*
CSHF - DEEP (CTRL)	0.1 °*C* (31%)	0.4 °*C* (11%)	0.1 °*C* (23%)	0.5 °*C* (24%)	0.2 °*C* (16%)	0.2 °*C* (20%)	**0.3** °*C* (**16%)**	0.2 °*C* (12%)	0.3 °*C* (148%)	0.6 °*C* (31%)	0.2 °*C* (13%)
WAVES - CTRL (CSHF)	0.3 °*C* (310%)	1.3 °*C* (310%)	0.5 °*C* (520%)	2.2 °*C* (420%)	0.0 °*C* (6%)	1.5 °*C* (610%)	**1.8** °*C* (**660%)**	1.4 °*C* (710%)	0.9 °*C* (360%)	3.2 °*C* (550%)	2.0 °*C* 850%)
WAVES - DEEP	130%	40%	140%	110%	15%	150%	**125%**	100%	680%	200%	120%

The last row WAVES-DEEP is representing the percentage enhancement due to join effects of waves and continental shelf compared to deepwater, no-wave experiments.

### Whitecapping induced acceleration, source of momentum

**Figure 3:**
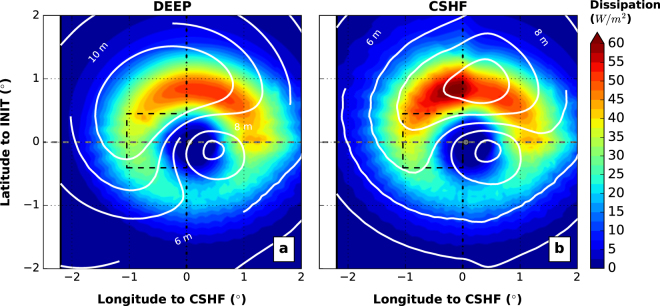
Total surface wave-induced energy dissipation just before the cyclone reaches the continental shelf (gray dashed line and circle represent the track and the cyclone eye, respectively). (**a**) For experiment *CTRL-DEEP* (no continental shelf). (**b**) Same for *WAVES-CSHF* experiment. Significant wave heights are shown with white contours (every 2 *m*). This highlights an enhancement of the wave-induced energy dissipation over the shelf linked to a reduction of significant wave heights.

**Figure 4:**
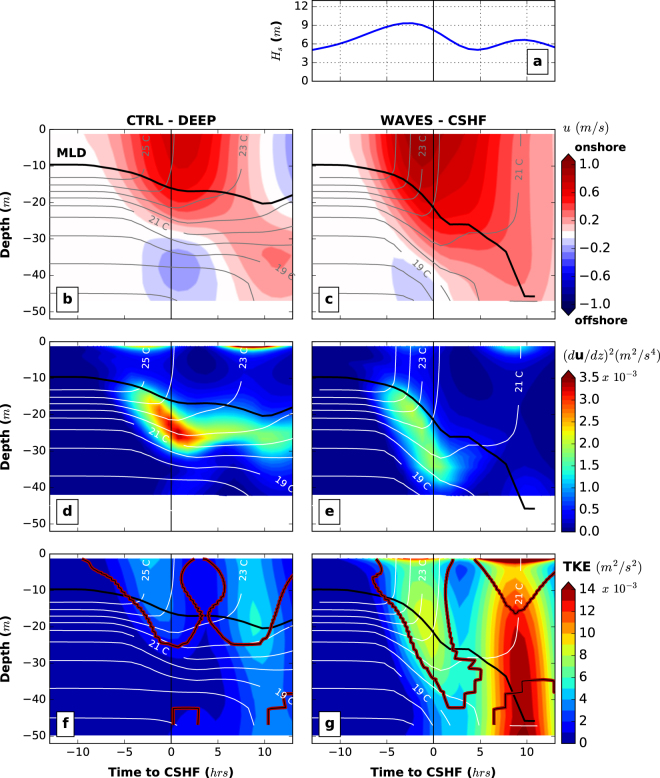
Impact of waves on the vertical mixing. (**a**) Significant wave height. Time series of the spatially-averaged cross-shore velocity (**b** and **c**), vertical current shear (**d** and **e**) and turbulent kinetic energy (**f** and **g**) depth-profile for the *CTRL-CSHF* and *WAVES-CSHF* experiments. Values are averaged over the same spatial box as shown in Fig. 2. MLD and 1 *°C* temperature contours are given in black solid line and white plain lines, respectively. The thin black line superimposed on a thick red line represents a Richardson number of 0.5 (in **f** and **g**). The x-axis 0 represents the time when the cyclone reaches the continental shelf. The cyclone characteristics and initial ocean state are the same as in Fig. 1.

To understand the key wave processes driving the SST cooling, each wave term in the continuity, momentum, tracer and turbulence equations were isolated separately. The only large driver of the extra cooling comes from the whitecapping-induced acceleration at the surface (**F**_**W**_ in equation 5, see Methods section). In the presence of a continental shelf, the dissipation induced by whitecapping increases by around 15–20%, while the maximum box-averaged significant wave heights diminishes 30% from 12 m to 9 m (Fig. 3). The wavelength decreases from around 300 m to 150 m and these different wave characteristics all participate in an increase of the whitecapping-induced acceleration. Vertical profiles of spatially-averaged cross-shore velocities (Fig. 4b,c) show a net increase of the onshore flow with strong velocities near the surface driven by the wave dissipation. The changes in the circulation above the continental shelf leads to weaker vertical current shear near the surface but stronger shear in the middle of the water column below the mixed layer depth (Fig. 4d,e). Therefore, vertical profiles of turbulent kinetic energy (TKE) reveal a strong enhancement by waves (Fig. 4f,g). Particularly Fig. 4f,g shows two peaks of strong TKE, one ahead-of-the-cyclone-eye (due to ahead winds) and a second one behind. This is correlated with the two modes of MLD deepening seen in Fig. 1. The TKE is over 3.10^−3^*m*^2^/*s*^2^ in the first 15–20 *m* under the surface for the *CTRL-CSHF* experiment. This contrasts sharply with the *WAVES-CSHF* experiment which shows the TKE with waves is larger than this threshold for most of the water column. While the strongest TKE is located close to the surface due to stronger wave dissipation (in the turbulence closure scheme), a second peak of TKE is now larger than in the *CTRL-CSHF* experiment in the middle of the water column (15–35 *m* water depth) linked to the increase of vertical shear of horizontal currents. When the cyclone approaches the continental shelf, the TKE becomes around five times stronger. The second peak (due to winds behind the cyclone) of TKE is systematically stronger than the first one. The winds ahead of the cyclone mix the ocean and deepen the MLD diminishing the stratification. Then the TKE associated with the winds behind the cyclone penetrates deeper due to a weakened stratification state of the ocean. The Richardson number (*Ri*) is smaller in these regions of mixing (Fig. 4f,g). The effects are not driven by the bottom stress. The key additional source in the momentum over the continental shelf is linked to the wave energy dissipation that drives stronger vertical mixing, redistributing the temperature between surface and bottom layers (Fig. 1d). A heat budget is presented in the Supplementary Figures S3 and S4 for each of the four experiments. It shows that the vertical and horizontal advections balance each other, but the vertical diffusion matches and drives the temperature changes rate.

### Sensitivity to ocean and TC properties

Our base case is similar to the real case study of Hurricane Irene in the mid-Atlantic Bight^16,17^. Wave states and the ocean response are sensitive to the initial cyclone and ocean characteristics. A set of experiments with varying maximum wind speed *V*_*max*_, radius of maximum winds *R*_*max*_, transitional speed *U*_*bck*_, incidence to the continental shelf for the atmospheric conditions, MLD, depth of the continental shelf and steepness of the vertical temperature profile (*Weak* or *Steep* - Supplementary Fig. S1b) for the ocean conditions are performed. Table 1 reveals that for all cases considered the waves have a large impact on the ahead-of-the-cyclone cooling. The presence of a continental shelf increases the cooling compared to the deep water case by only 0.2–0.5 *°C*. However the cooling induced by waves (still in the presence of a continental shelf) is 3–7 times stronger than the enhancement due to the continental shelf itself with additional cooling reaching up to 3 *°C*. Overall, compared to the *CTRL-DEEP* experiments, this additional cooling is at least as large as the direct cooling induced by only the cyclone winds.

For fast moving cyclones (Exp. 5 in Table 1), the effect of waves on the ocean cooling is weaker as the TC stays a shorter duration over the continental shelf. Stronger winds (Exp. 2) drive stronger circulations and therefore mixing which can penetrate and alter deeper layers of the water column leading to significant reduction of the SST. In these circumstances, additional wave-induced cooling appears less important (40%) but still remains large (about 1.5 *°C*). When the MLD is larger (Exp. 9), cyclone-driven winds are not able to break and mix the stratified ocean. However the wave-driven processes can affect deeper layers of the water column enhancing the cooling by about a factor of 7 equivalent to 1.2 *°C*.

### Coupled Atmosphere-ocean-wave simulations

We have shown that cyclone-driven waves can have a large impact on the coastal ocean and the ahead-of-the-cyclone cooling. Enhanced cooling should lead to weaker winds and smaller waves therefore reducing the wave-induced impact and cooling. Fully-coupled experiments can be used to explore the net impact of wave-induced ahead of storm coastal cooling. However we acknowledge the fact that the wave to atmosphere coupling (surface roughness or sea spray, for example; not accounted here) is a positive feedback and can compete against the negative feedback induced by waves^13^.

**Figure 5:**
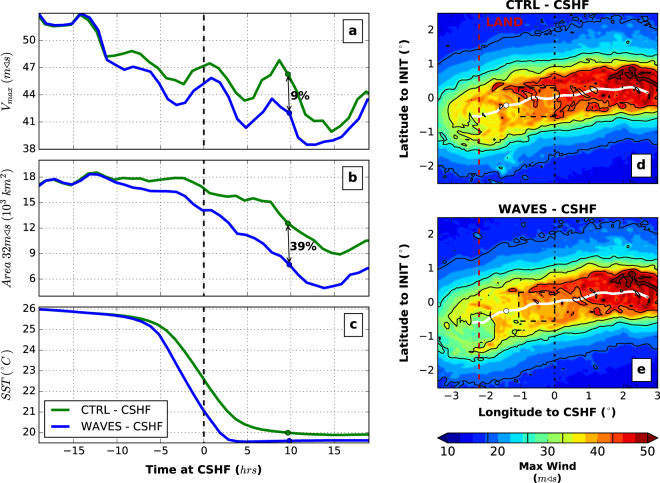
Fully-coupled atmosphere-ocean-wave experiments. (**a**) Time evolution of *V*_*max*_ for the *CTRL-CSHF* (green) and *WAVES-CSHF* (blue), respectively. (**b**) Same but for area where winds are stronger than 32 *m*/*s* (hurricane threshold). (**c**) Spatially-averaged SST in the dashed box displayed in (**d**) and (**e)**. (**d**) Maximum wind footprint aggregated over time for experiment *CTRL-CSHF*; the cyclone track is displayed in white plain line. (**e**) Same as (**d**) but for experiment *WAVES-CSHF*. The circle in each figure represents a point when the cyclone travels over the continental shelf before landfall; landfall is about 16 h after reaching the shelf. As for the previous figures, the x-axis 0 represents the time when the cyclone reaches the continental shelf.

In these coupled experiments, the ahead-of-cyclone cooling is enhanced in the presence of waves by around 1.5–2 *°C* (Fig. 5c) and the cyclone Vmax is reduced by about 5–10% (Fig. 5a). The regions with hurricane winds (larger than 32 *m*/*s*) are significantly reduced before landfall by up to 50% in the presence of waves (Fig. 5b and Supplementary Fig. S6). The integrated kinetic energy (IKE) and the integrated power dissipation (IPD) have a strong relationship with damages^40–42^ and are, in this experiment, reduced by about 10–15% and by up to 20% in the presence of waves, respectively (Supplementary Fig. S6). The largest IKE and IPD differences occur after the cyclone crosses the continental shelf (2–10 hours). Similar results are obtained using different initial SST (from 26 to 30 *°C*), different initial MLD (from 10 to 25 *m*) and different spin-up duration (from 7 to 9 days). Increasing the wind speed threshold leads to smaller regions and obviously larger percentage differences between *CTRL-CSHF* and *WAVES-CSHF* experiments.

## Discussion

Air-sea interactions play a key role in TC thermodynamics and SST changes can rapidly and significantly impact TC intensity^5,9–12^. Waves are the interface between the atmosphere and the ocean, implying feedbacks on both fluids through the transfer of momentum, mass and heat^24–26,43^. Recent studies^3,13,23,30,44^ have investigated the impact of particular wave-induced processes (sea spray, surface roughness, mixing, among others) on TC characteristics through fully-coupled atmosphere-ocean-wave models but are limited to historical case studies or have not studied the nearshore continental shelf impact. While some studies includes wave-current interactions^13,44^, the wave effects is commonly limited to an enhancement of the shear production^23^ or directly the mixing coefficients^3^. Here we find significant wave-current interactions and an impact of wave-driven circulations on the mixing. The transfer of energy due to wave dissipation at the edge of a continental shelf is able to drive stronger mixing and SST cooling.

Using a series of idealized simulations where cyclone and ocean properties are changed, the importance of wave-induced mixing in TC evolution has been demonstrated to be robust for coastal stratified oceans. Therefore a new mechanism, of whitecapping-induced acceleration as a negative feedback for tropical cyclones in coastal ocean is proposed. The wind-only experiments show that the presence of a continental shelf itself only slightly enhances the ahead-of-cyclone-eye cooling compared to deep water while other recent studies found a continental shelf effect on cooling^16,17^. However, in these studies they did not test for the null hypothesis of what would happen in an equally stratified deep ocean, instead they only examined a continental shelf. Our experiments show that for similarly stratified deep water one can expect similar wind-driven ahead of cyclone cooling and current shear as in presence of a continental shelf. However the change of cyclone-induced wave heights due to strong energy dissipation (whitecapping) on the continental shelf increases the mean flow driving deeper vertical shear which drastically enhances the vertical mixing in the ocean. This mixing propagates through the water column, approximately following a buoyancy gradient. This leads to a strong cooling of the surface and a warming of the bottom layers.

Our results corroborate the key impact of the upper ocean stratification in determining the strength of the coupled feedback^45^, but they also highlight that wave-current interactions and induced mixing can reach deeper water and therefore enhances the negative feedbacks compared to atmosphere-ocean only coupling. This is a property of the coastal shelf not found in deep water. The effects of the cyclone winds on the coastal ocean can be more than doubled when including to wave-induced processes. This study is based on idealised coupled experiments in order to explore a range of cyclone and ocean conditions instead of simulating a particular historical case study, and we acknowledge the fact that no tuning has been undertaken to fit any observation. Using different formulations can impact the magnitude of the cooling^23^, but we show here than in coastal area where strong steep locally-generated waves might occur, they can be crucial to capture the response of the ocean.

In contrast to the atmosphere, the ocean recovery is slow^28^ and deeper layers can take weeks to re-adjust after a strong TC. Reduced stratification may promote the intensity of following cyclones. There are therefore some interesting additional implications for multiple events as for example Hurricanes Gustav and Ike (2008) or Typhoons Chaba and Songda (2004) with similar tracks within a one week window. Further investigations are required to understand the wider ranges of ocean and atmosphere conditions to fully characterise the wave impacts. However this study clearly shows non-negligible effects of wave-induced mixing in coastal stratified regions which will have implications on highly vulnerable property and people.

## Methods

### ROMS model

The ocean is simulated using the regional free-surface, *σ*-coordinate, primitive equation model ROMS (Regional Ocean Modelling System^34^, svn 820). Two idealised ocean setups are considered here (both including land on the West side): a constant bathymetry (set to 500 *m*) while the second setup includes an about 200 *km*– wide, 50 *m*– deep continental shelf connecting the land to the deep water regions (Supplementary Fig. S1a). The connection from the continental shelf to the deep ocean consists of a smoothed (on the edge) linear steep slope over 150 *km*. They are denoted *DEEP* and *CSHF*, respectively. 40 vertical levels are used with increasing resolution near the surface.

The initial state of the ocean is spatially uniform with no currents or surface elevation. The temperature and salinity profiles are from the World Ocean Atlas^46,47^ averaged over July-August for the North West Atlantic (Supplementary Fig. S1b). The temperature profile is tuned and shifted in order to increase the steepness in the surface layers to create a strong near-surface stratification which is in the range of observations on the Atlantic Bright^16^ (denoted *Weak*). In the second temperature profile an even larger gradient is used for sensitivity analysis (dashed line in Fig. 1b, denoted *Steep*). The initial SST is set at 26 *°C*. The MLD is about 10 *m*. The boundaries are open (except the western boundary set as land) with a nudging for the tracers towards the initial conditions. The Coriolis parameter is constant (8.3 10^−5^*s*^−1^ for 35 *N*^16^). A sponge layer of 20 elements is applied on each side and a 20*s* time step is used. An ocean-only run is carried out to obtain an adjusted temperature/salinity profile. In order to study only the impact of the winds and pressure, all other fluxes from the bulk flux formulation are turned off in the ocean-only and ocean-wave experiments.

### SWAN model

The third generation wave model SWAN^38^ solves the wave action balance equation, modelling the wind-growth, the non-linear transfer of energy and the dissipation of energy by breaking, shallow water effects and bottom friction. The spectral grid consists of 36 directions (10° resolution) and 25 frequencies (corresponding to periods varying logarithmically from 2 to 25 *s*). SWAN runs in non-stationary mode with a 5*min* time step and is only forced by winds at 10 m. Feedbacks of surface elevation, depth-averaged currents and bottom roughness are provided by ROMS. Default SWAN nonlinear wave-wave interactions (triads and quads) are activated. Dissipation by whitecapping and depth-induced breaking as well as by bottom friction are taken into account but no sensitivity has been undertaken for the different parameterisations used and default SWAN parameters are used here. The initial state consists of a wave-free ocean and it takes around 12 simulated hours to built substantial waves (around 12 m wave height) near the cyclone core.

### Ocean-Wave coupling

The default version of the Coupled-Ocean-Atmosphere-Wave-Sediment Transport Modeling System^36^ (COAWST) is used to represent the complex three-dimensional wave-current interactions according to the Vortex Force approach^37,48^. It includes wave-induced currents and the injection of wave-induced mixing at the surface. Defining *z* the vertical component (and $\hat{{\bf{z}}}$ the 3D vertical unit vector) while the other bold-type characters represent the horizontal components, and ⊗ the outer product and × the curl operator, the conservation of mass and momentum is written as follows^39^:

1 $$\nabla \mathrm{.}{{\bf{u}}}^{L}+\frac{\partial {w}^{L}}{\partial z}=0$$

2 $$\begin{array}{ll}\frac{\partial {\bf{u}}}{\partial t} & +\,\nabla \mathrm{.}({\bf{u}}\otimes {\bf{u}})+\frac{\partial (w\,{\bf{u}})}{\partial z}-{{\bf{F}}}_{{\bf{BF}}}-{{\bf{D}}}_{{\bf{m}}}+\frac{\partial }{\partial z}(\overline{{\bf{u}}{\boldsymbol{^{\prime} }}w^{\prime} }-{\nu }_{m}\frac{\partial {\bf{u}}}{\partial z})-{{\bf{F}}}_{{\bf{C}}{\bf{u}}{\bf{r}}{\bf{v}}{\bf{i}}}^{{\bf{L}}}\\  & +\,f\mathrm{.}\hat{{\bf{z}}}\times {{\bf{u}}}^{L}+{\bf{u}}\,\frac{\partial {w}_{S}}{\partial z}+{\bf{u}}(\nabla {\boldsymbol{.}}{{\bf{u}}}_{{\bf{s}}})=-\,\hat{{\bf{z}}}\times {{\bf{u}}}_{{\bf{S}}}(\hat{{\bf{z}}}\mathrm{.}\nabla \times {\bf{u}})-{w}_{S}\frac{\partial {\bf{u}}}{\partial z}+{{\bf{F}}}_{{\bf{W}}}-\nabla \mathrm{.}{{\varphi }}^{c}\end{array}$$

3 $$\frac{\partial {\varphi }^{c}}{\partial z}+\frac{g\rho }{{\rho }_{0}}={{\bf{u}}}_{{\bf{S}}}\mathrm{.}\frac{\partial {\bf{u}}}{\partial z}$$

where (**u**^*L*^, *w*^*L*^) represents the Lagrangian velocities, sum of the “quasi”-Eulerian mean flow (**u**, *w*) and the Stokes drift (**u**_*S*_, *w*_*S*_). *g* is the gravity. *ρ* and *ρ*_0_ represent the density and mean density, respectively. *ϕ*^*c*^ is the sum of the dynamic pressure and Bernouilli head. **F**_**BF**_, **D**_**m**_ and **F**_**W**_ represent the non-wave non-conservative force, the parameterised momentum horizontal mixing term and the non-conservative dissipative wave forcing, respectively. (**u**′, *w*′) are the Reynolds turbulent velocities, ν_*m*_ the momentum viscosity and the Coriolis parameter is given by *f*. Due to the orthogonal curvilinear grid, corrections of the advective terms occur in ${{\bf{F}}}_{{\bf{Curvi}}}^{{\bf{L}}}$ and depend on the Lagrangian velocities^37^. Finally, the Stokes drifts is expressed as:

4 $${{\bf{u}}}_{{\bf{S}}}=\frac{{c}_{w}{E}_{w}}{g}\,\frac{cosh(2{k}_{w}[z+h])}{sin{h}^{2}({k}_{w}D)}{{\bf{k}}}_{{\bf{w}}}\,{\rm{and}}\,{w}_{S}=-\,\nabla \mathrm{.}{\int }_{-h}^{z}{{\bf{u}}}_{{\bf{S}}}\,dz$$

with ∇ the del operator, *h* is the bathymetry while *D* represents the wave-averaged thickness of the water column. *c*_*w*_ and *E*_*W*_ are the phase velocity and the wave energy, respectively. **k**_**w**_ is the wave number. The non-conservative wave forcing ***F***_***W***_ represents the acceleration due to whitecapping and depth-induced breaking^37,49^ (*ε*_*w*_, their dissipation and *H*_*rms*_ is the root mean square wave height):

5 $${{\bf{F}}}_{{\bf{W}}}=\frac{{\varepsilon }_{w}}{{\rho }_{0}\,\sigma }{k}_{w}\frac{FB}{{\int }_{h}^{{\varphi }^{c}}FBdz}\,with\,FB=cosh(\frac{2\pi }{{H}_{rms}}[z+h])$$

The tracer conservation (used for the heat budget) is written as:

6 $$\frac{\partial c}{\partial t}+\nabla \mathrm{.}({{\bf{u}}}^{L}c)+\frac{\partial ({w}^{L}c)}{\partial z}-{C}_{src}+\frac{\partial }{\partial z}(\overline{c^{\prime} w^{\prime} }-{\nu }_{c}\frac{\partial c}{\partial z})=0$$

with *c* representing any tracer concentration, *ν*_*c*_ the tracer diffusivity and *C*_*src*_ consists of the tracer source/sink terms. The viscosity and diffusion terms are computed according to the Generic Length Scale k-*ω* model for transport of kinetic energy^50,51^:

7 $$\begin{array}{ccc}\frac{{\rm{\partial }}k}{{\rm{\partial }}t}+{\bf{u}}.{\rm{\nabla }}k & = & \frac{{\rm{\partial }}}{{\rm{\partial }}z}(\frac{{K}_{M}}{{\sigma }_{k}}\frac{{\rm{\partial }}k}{{\rm{\partial }}z})+P+B-\varepsilon \\ \frac{{\rm{\partial }}\omega }{{\rm{\partial }}t}+{\bf{u}}.{\rm{\nabla }}\omega  & = & \frac{{\rm{\partial }}}{{\rm{\partial }}z}(\frac{{K}_{M}}{{\sigma }_{\omega }}\frac{{\rm{\partial }}\omega }{{\rm{\partial }}z})+\frac{\omega }{k}({c}_{w1}P+{c}_{w3}B-{c}_{w2}\varepsilon )\end{array}$$

with *k* and *ω* the turbulent kinetic energy and the rate of dissipation of energy per unit volume and time, respectively. *K*_*M*_ is the eddy viscosity for momentum related to the Reynolds stress tensor $\overline{{\bf{u}}{\boldsymbol{^{\prime} }}w^{\prime} }=\,-{K}_{M}\frac{\partial {\bf{u}}}{\partial z}$ . *ε* is a dissipative term. *P* and *B* represent the production of turbulence by shear and buoyancy, respectively. The coefficients (σ_*_, *c*_w*_) are set to their constant default value. The wave energy dissipation is injected at the surface as ${\frac{{K}_{M}}{{\sigma }_{k}}\frac{k}{z}|}_{\zeta }={\varepsilon }_{w}$ where *ε*_*w*_ is the downward TKE flux due to wave energy dissipation by both whitecapping and depth-induced breaking and *ζ* is the free surface. Similarly the wave dissipation is included in the surface forcing for the rate of dissipation of energy *ω* (Equations 44–45^37^).

### Ocean-only and Ocean-Wave coupled experiments

The computational domain extends for around 25° in the West-East direction and 18° in North-South with an average horizontal resolution of 5 km. The same grid is used in SWAN to generate, propagate and transform the wave fields. SWAN and ROMS exchange information every 10 simulated minutes. The wind field is generated with the analytic TC wind profile model^35,42^ (*λ*-model) describing the wind profile of TCs in terms of *V*_*max*_ and *R*_*max*_ (Supplementary Fig. S2a) combined with a constant background flow. The control TC consists of *V*_*max*_ = 35 *m*/*s*, *R*_*max*_ = 100 *km*, *U*_*bck*_ = 5 *m*/*s*. A set of independent experiments were carried out to investigate the impact of the wind field (*V*_*max*_, *R*_*max*_, *U*_*bck*_ and traveling direction) on the ahead-of-the-cyclone cooling as well as the influence of the ocean characteristics (depth of the continental shelf, MLD, temperature profile steepness and latitude for Coriolis parameter); see Table 1. Each experiment is run with and without the presence of a continental shelf and with wave-induced dynamics either activated or not.

### Sensitivity analysis to modelled physical processes

Many parameterisations have been proposed to represent wave-current interactions and are based on different tuned parameters. The aim of this study is to investigate a new physical mechanism through an idealised set-up in order to explore a range of ocean and tropical cyclones conditions. Therefore, no tuning or complex formulations have been integrated here but we acknowledge that our results and the intensity of the SST cooling may vary quantitavely depending on the ocean-wave parameterisation implemented. No additional shear production is considered here^23^. The effects of Langmuir circulations are not activated and roller effects, bottom and surface streaming are not considered. Only the effect of wave-induced currents, the injection of wave energy at the surface and the presence of a continental shelf are investigated in terms of SST cooling ahead-of-the-cyclone. The enhancement of eddy viscosity due to waves^39^ has been tested and leads to additional SST cooling underlining that our results are conservative.

Without depth-induced breaking at the edge of the shelf the TKE slightly reduces. However, the same behaviour is seen so that it can be concluded that surface cooling is not induced by depth-induced breaking (as expected). Similarly with no bottom friction in SWAN (very small roughness length) the response remained the same (not shown). Different expressions for the vertical distribution function *FB* could be used^37,49^ while other studies suggest that the whitecapping should only act on the surface layer. Changing the vertical distribution in our set-up leads to similar results and applying the whitecapping acceleration (equation 5) in the surface layer only also shows similar results but with smaller surface cooling (see Supplementary Figure S5). Finally, different coefficient for determining the rate of whitecapping dissipation in SWAN (1.36e-5 to 3.36e-5, default 2.36e-5) and different wave steepness for a Pierson-Moskowitz spectrum (2.02e-3 to 4.02e-3, default 3.02e-3) were experimented and also lead to similar results (not shown).

### WRF model

The atmosphere is simulated with the widely-used Advance Research Weather Research and Forecasting atmospheric model^52^ (AR WRF 3.7.1). The WRF single-moment 6-class microphysics scheme^53^ is used while the Mellor-Yamada-Janjic planetary boundary layer scheme (MYJ)^54^ is combined with the Eta similarity surface layer scheme^55^. Long- and short-wave radiations are computed using the Rapid and accurate Radiative Transfer Model^56^ (RRTM). However, the shortwave radiation is tuned to be uniform spatially and temporally, and to compensate the cooling induced by the long-wave emission so that the averaged SST over the domain does not decrease over time. The cumulus scheme is turned off in the present high-resolution setup (5 *km*). WRF is also run on a 35*N* f-plane with a 40 *s* time step, 31 vertical levels and periodic boundary conditions in the West-East direction. Even with the presence of land, this periodic condition minimizes boundary effects and leads to unperturbed cyclones in the region of development.

### Coupled Atmosphere-Ocean-Wave experiments

The atmosphere-ocean-wave experiments are on a domain twice as the ocean-only simulations. The horizontal resolution is maintained at around 5 *km*. ROMS and WRF share the same grid while SWAN runs on a 10−*km* grid for performance purpose. WRF passes latent and sensible heat fluxes, wind stresses and surface roughness, 2 *m* temperature, mean sea level pressure (MSLP), relative humidity, rain and evaporation rates to ROMS. WRF SST is updated from ROMS. Winds are provided to SWAN but the wave-induced surface roughness is not accounted for in this study. WRF, ROMS and SWAN exchange information every 10 simulated minutes.

A 7.5− day atmosphere-only over-ocean static spin-up is carried out to develop a realistic cyclone (Supplementary Fig. S2b) from the analytic wind profile used previously. This becomes the initial condition of the fully-coupled atmosphere-ocean-wave experiments. Transitional speed and land are added after the spin-up period. This mature idealized cyclone travels for two days towards the coast crossing the continental shelf with a normal incident direction and a transitional speed of 5 *m*/*s* with a similar SST as in the previous experiments (26 *°C* leading to a *V*_*max*_ of around 45 *m*/*s* over the continental shelf). To maintain the approaching cyclone winds similar to the uncoupled experiments the MLD over the continental shelf is about 20 *m* while the deep ocean MLD is set to around 30 *m* in order to prevent stronger winds and instabilities away from the cyclone to mix the ocean before the cyclone approaches the area of interest. A range of runs have been considered: initial SST ranging from 26 to 30 *°C*, different spin-up duration and different initial MLD; all showing similar behavior (not shown).

### Post-Processing

Time series of various properties are spatially-averaged in an about 100−*km* square box over the continental shelf, starting at the edge of the shelf (dashed line in Fig. 2b and d). The time reference is set when the cyclone crosses the continental shelf (linear interpolation) and enters the box; therefore negative time means ahead-of-the-cyclone-eye. Instantaneous snapshots (Figs 2b,d and 3) are given at the last time step before the cyclone enters the continental shelf (cyclone eye is marked as a circle). For the sensitivity analysis, only the differences for the ahead-of-the-cyclone cooling is considered. In Supplementary Figure S6, IKE and IPD are computed according to previous studies^40–42^. All figures have been generated using standard Python libraries.

### Data availability

Model inputs and outputs used for the present study are available from the corresponding author on request.

## Electronic supplementary material

Supplementary Information
