# Publisher Correction: Impact of wave whitecapping on land falling tropical cyclones

**DOI:** 10.1038/s41598-018-24117-4

**Published:** 2018-04-17

**Authors:** Nicolas Bruneau, Ralf Toumi, Shuai Wang

**Affiliations:** 1 Blackett Laboratory, Department of Physics, Imperial College, Prince Consort Road, London, SW7 2AZ UK; 2 Present Address: National Oceanography Centre, Joseph Proudman Building, Liverpool, L3 5DA UK

Correction to: *Scientific Reports* 10.1038/s41598-017-19012-3, published online 12 January 2018

This Article contains errors in References 8, 9, 10, 17 and 48, which were incorrectly given as:

8. Schade, L. R. & Emanuel, K. A. The ocean’s effect on the intensity of tropical cyclones: Results from a simple coupled atmosphere-ocean model. *Journal of the Atmospheric Sciences* **56**, 642–651, 10.1175/1520-2840469 (1999).

9. Chan, J. C. L., Duan, Y. & Shay, L. K. Tropical cyclone intensity change from a simple ocean-atmosphere coupled model. *Journal of the Atmospheric Sciences* **58**, 154–172, 10.1175/1520-0469 (2001).

10. D’Asaro, E. A. The ocean boundary layer below hurricane dennis. *Journal of Physical Oceanography* **33**, 561–579, 10.1175/1520-0485 (2003).

17. Seroka, G. *et al*. Stratified coastal ocean interactions with tropical cyclones. *Monthly Weather Review* **144**, 3507–3530, 10.1175/JAS-D-16-0100.1 (2016).

48. Uchiyama, Y., McWilliams, J. & Shchepetkin, A. Wave-current interaction in an oceanic circulation model with a vortex-force formalism: Application to the surf zone. *Ocean Modelling* **34**, 10–35, 10.1016/j.ocemod.2007.04.002 (2010).

The correct references are listed below as Refs^1–5^.
