# Supplementary material for: Impact of wave whitecapping on land falling tropical cyclones
Source: Sci Rep. 2018 Jan 12;8:652. doi: 10.1038/s41598-017-19012-3 (PMC5766526; doi:10.1038/s41598-017-19012-3)
Supplement: Supplementary file 1 — Supplementary Information [file 41598_2017_19012_MOESM1_ESM.pdf]

Supplementary Information

Impact of wave whitecapping on land falling tropical cyclones

Nicolas Bruneau[1,2,*], **Ralf Toumi**[1], **and Shuai Wang**[1]

[1]Blackett Laboratory, Department of Physics, Imperial College, Prince Consort Road, London SW7 2AZ, UK

[2]now working at National Oceanography Centre, Joseph Proudman Building, Liverpool L3 5DA, UK

[*]bruneau.n@gmail.com

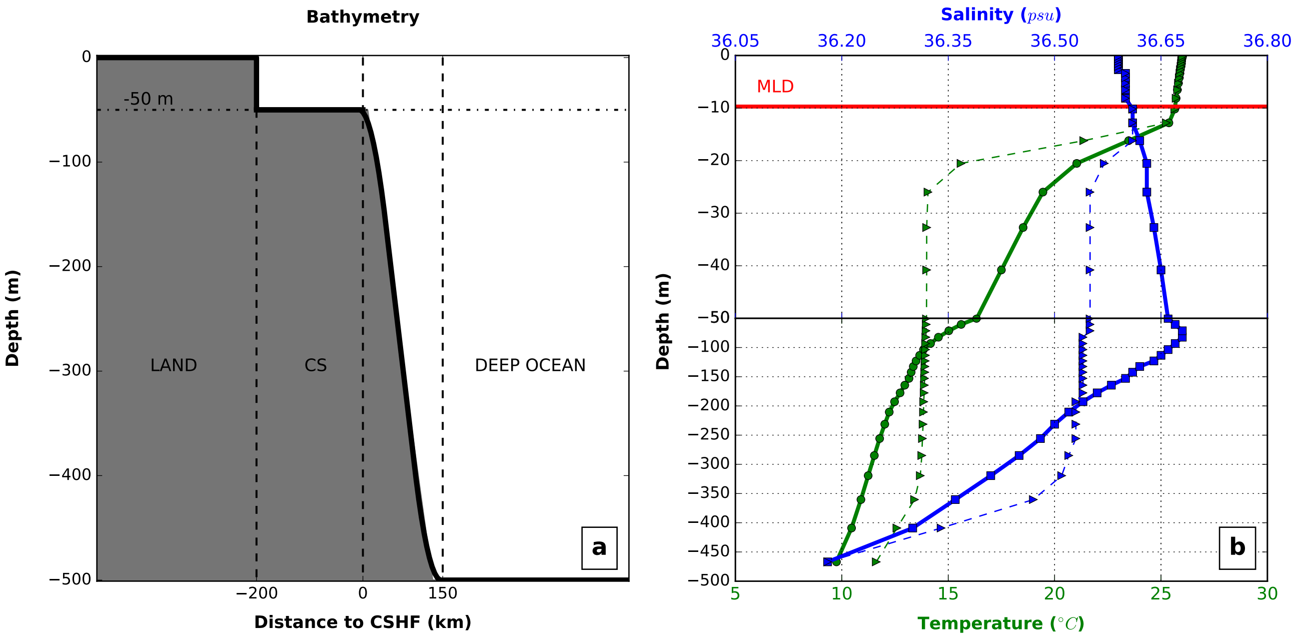

Supplementary Fig. S1. Ocean model description. (**a**) The idealised bathymetry with an about $200km-$wide, $50m$-deep continental shelf. (**b**) Idealised spatially constant vertical profiles of salinity (blue) and temperature (green). The dashed green line represent an alternative temperature profile with a steeper gradient. The thick red line illustrate the initial 10 m mixed layer depth.

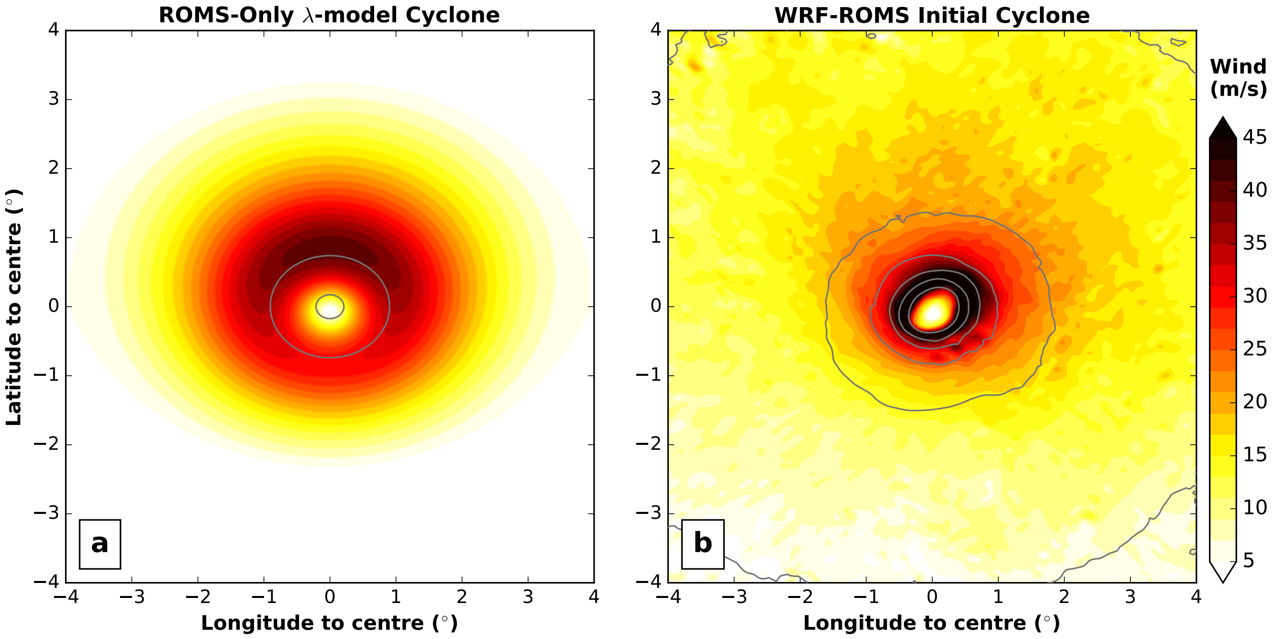

Supplementary Fig. S2. Atmosphere initial cyclones. (**a**) An example of idealised cyclone generated with the λ model ($V_{max} = 35\ m/s$, $R_{max} = 100\ km$ and $U_{bck} = -5\ m/s$). (**b**) For the coupled atmosphere-ocean-waves experiments where the cyclone is generated from a 7.5-day atmosphere-only static simulation using the analytic λ model as initial atmospheric perturbation combined with a 5 m/s transitional speed.

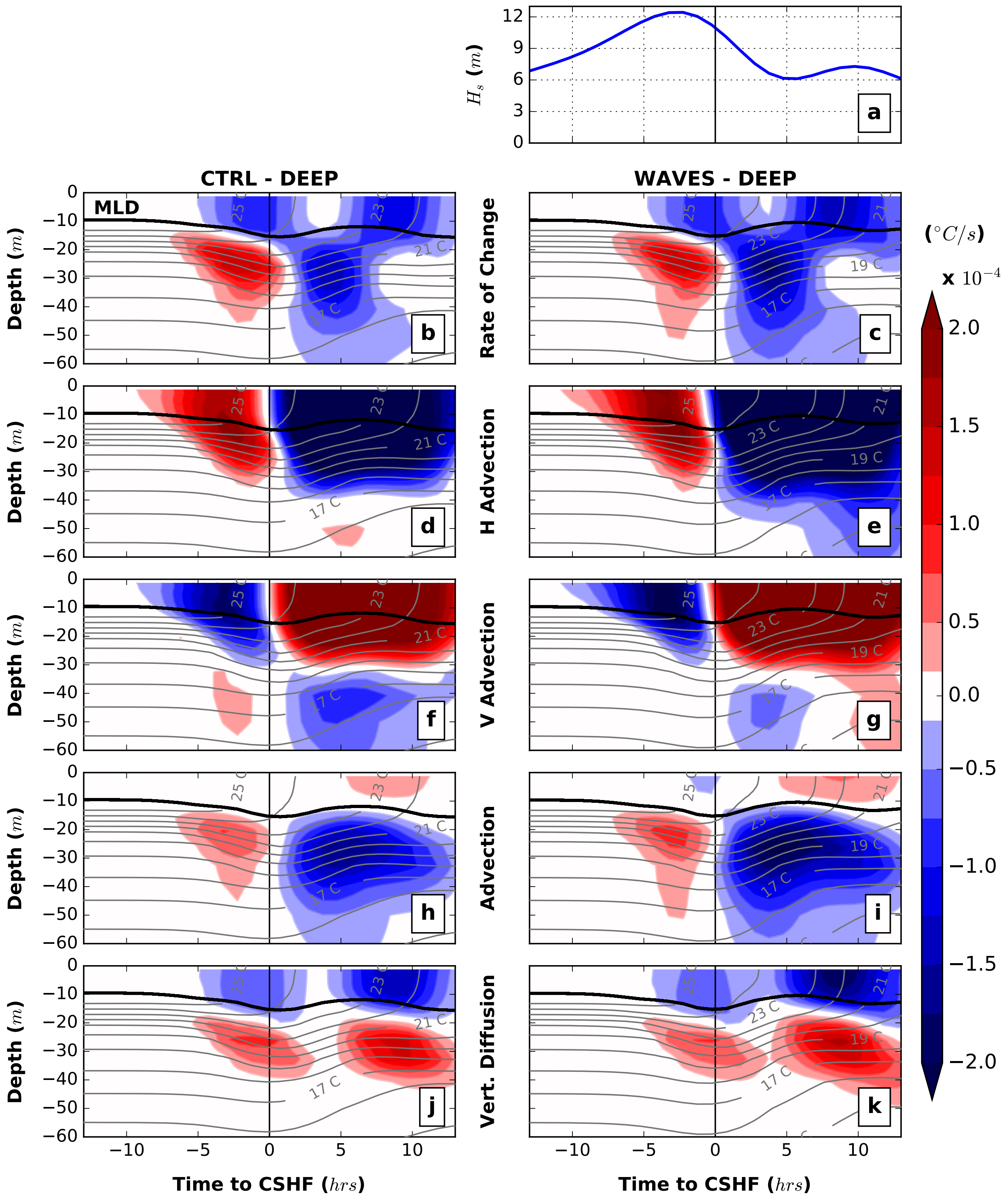

Supplementary Fig. S3. **Heat Budget for the deep water experiments**. Left panels: deep water experiment with no waves (*CTRL-DEEP*); right panels: deep water experiment with waves (*WAVES-DEEP*). (**a**) Box-averaged significant wave height. The rate of temperature change is given in (**b**) and (**c**). (**d**) and (**e**) show the horizontal advection while the vertical advection is given in (**f**) and (**g**). Finally the total advection and the vertical diffusion are provided in (**h**) and (**i**), and (**j**) and (**k**), respectively.

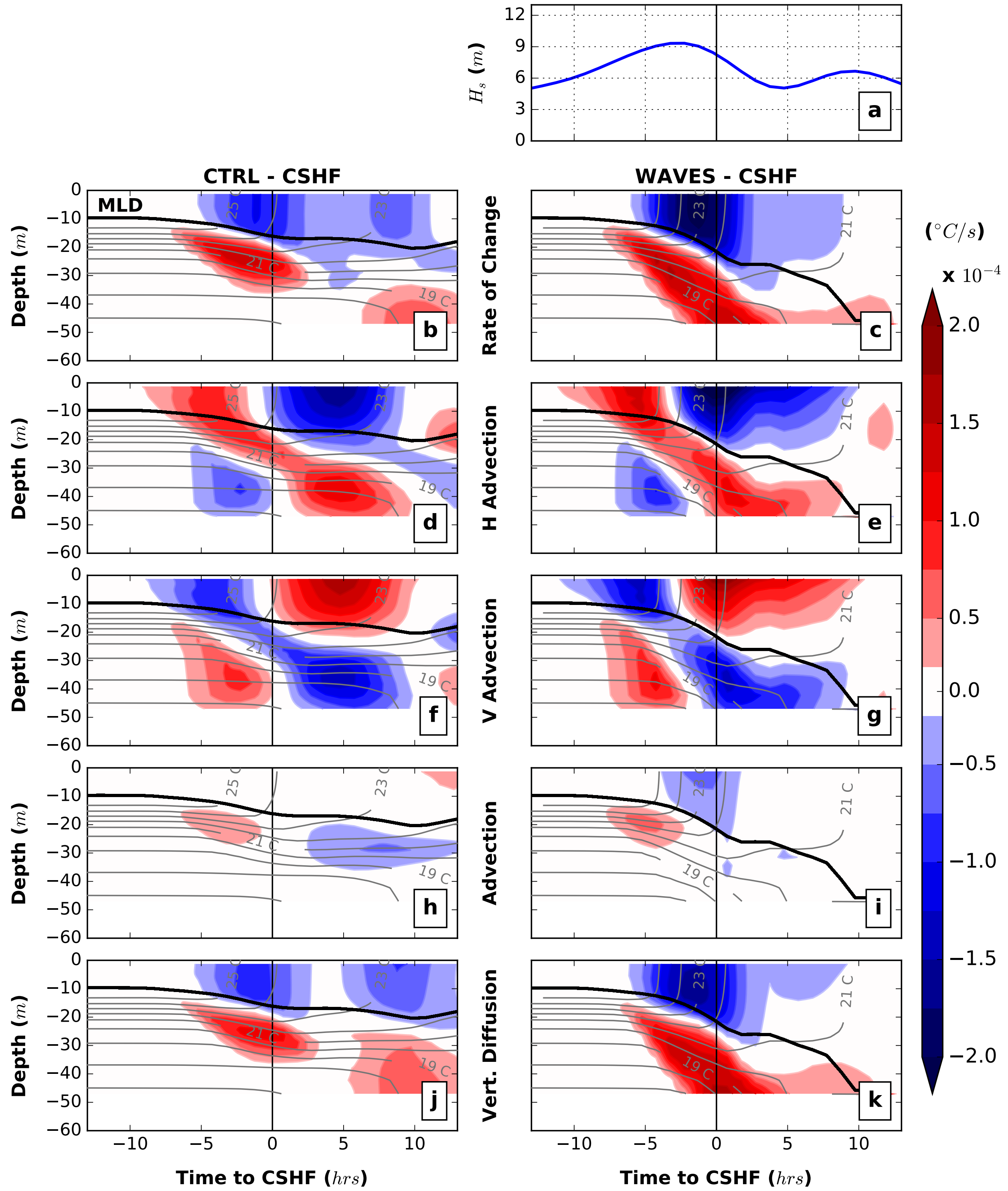

Supplementary Fig. S4. **Heat Budget for the continental shelf experiments**. Same as Supplementary Figure 3 but for *CTRL-CSHF* (left) and *WAVES-CSHF* (right) experiments.

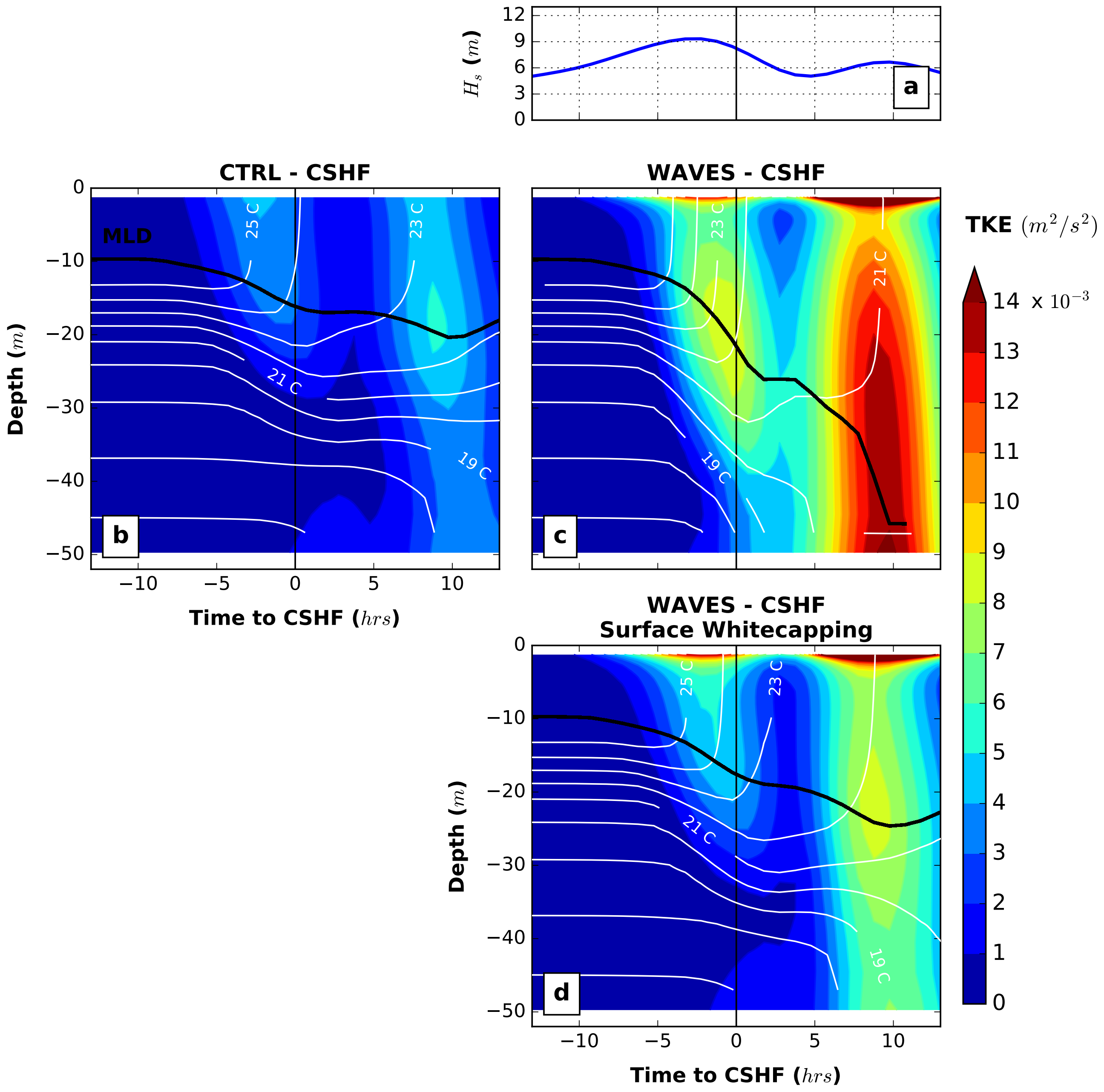

Supplementary Fig. S5. Effect of whitecapping vertical distribution on TKE (**a**) Significant wave height. Time series of the spatially-averaged turbulent kinetic energy for *CTRL-CSHF* (**b**), *WAVES-CSHF* with default vertical distribution for whitecapping (**c**) and *WAVES-CSHF* with total whitecapping dissipation injected in surface layer only (**d**). Values are averaged over the same spatial box as shown in Figure 2. MLD and $1°C$ temperature contours are given in black solid line and white plain lines, respectively. The x-axis 0 represents the time when the cyclone reaches the continental shelf. The simulations were carried out for an idealized analytical temporally-constant wind field characterized by a maximum wind speed $V_{max} = 35\ m/s$, a radius of maximum wind $R_{max} = 100\ km$, a transitional speed $U_{bck} = 5\ m/s$ and an ocean state with a $MLD = 10\ m$ with the *Weak* temperature profile

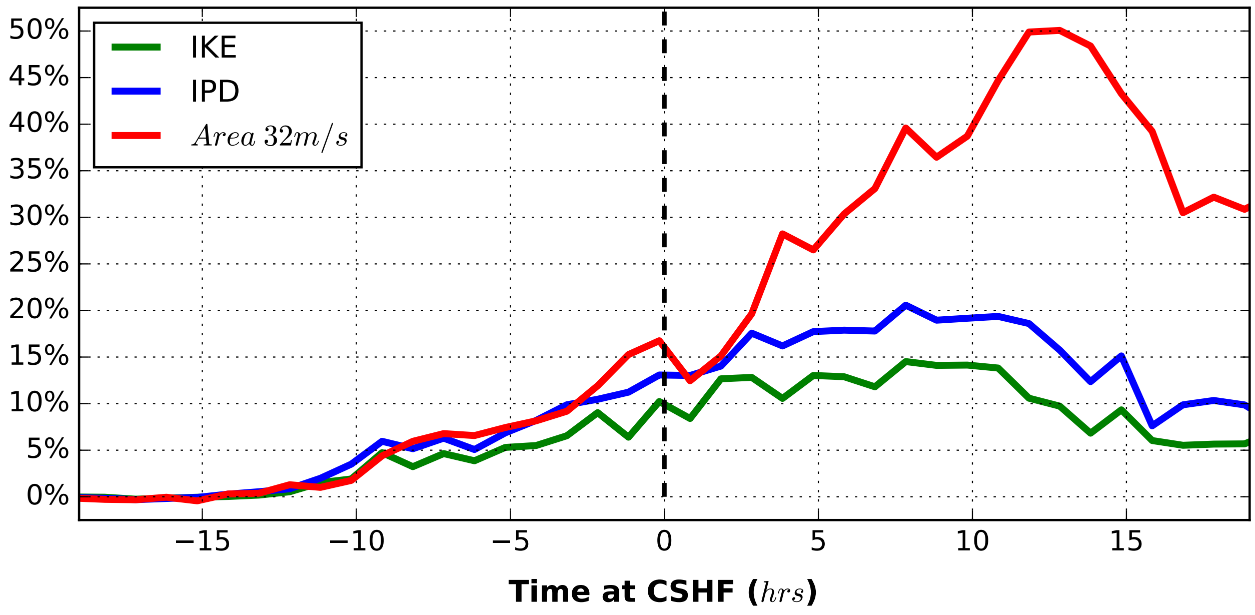

Supplementary Fig. S6. Percentage reduction of main metrics due to waves. Time series of percentage reduction between the two experiments (*CTRL-DEEP* and *WAVES-CSHF*) for: areas with winds higher to 32 m/s (red), IKE - Integrated Kinetic Energy (green) and IPD - Integrated Power Dissipation (blue). The x-axis 0 represents the time when the cyclone reaches the continental shelf. Landfall occurs about 16 h after reaching the shelf.
